# Correlates of disordered eating and insulin restriction behavior and its association with psychological health in Taiwanese youths with diabetes mellitus

**DOI:** 10.1186/s40337-023-00888-8

**Published:** 2023-09-14

**Authors:** Wei-Chih Chou, Yen-Yin Chou, Yu-Wen Pan, Tsung-Ying Ou, Meng-Che Tsai

**Affiliations:** 1 Department of Pediatrics, Hualien Tzu Chi Hospital, Buddhist Tzu Chi Medical Foundation, Hualien, Taiwan; 2 Division of Genetics, Endocrinology, and Metabolism, Department of Pediatrics, National Cheng Kung University Hospital, College of Medicine, National Cheng Kung University, Tainan, Taiwan; 3 Department of Genomic Medicine, National Cheng Kung University Hospital, College of Medicine, National Cheng Kung University, Tainan, Taiwan; 4 Department of Pediatrics, Dalin Tzu Chi Hospital, Buddhist Tzu Chi Medical Foundation, Dalin, Taiwan; 5 Department of Medical Humanities and Social Medicine, College of Medicine, National Cheng Kung University, 138 Shengli Road, Tainan, 704 Taiwan; 6 Department of Pediatrics, Shin Huey Shin Hospital, Kaohsiung, Taiwan

**Keywords:** Diabetes mellitus, Disordered eating behavior, Insulin restriction, Depression, Anxiety

## Abstract

**Background:**

Adolescents and young adults (AYAs) with diabetes mellitus (DM) are prone to eating disorders that may worsen metabolic control. This study investigated the clinical and behavioral correlates of disordered eating and insulin restriction (DE/IR) behavior and its association with psychological health among AYAs with DM.

**Methods:**

We enrolled patients with DM aged 10–30 years receiving insulin treatment in a tertiary medical center from 2019 to 2021. After obtaining informed consent, we assessed various visit-to-visit HbA1c measures indicating glycemic control, DE/IR behavior using the modified SCOFF questionnaire, weight-control practices (e.g., self-medication, induced vomiting, and over-exercising), and anxious and depressive symptoms using the Hospital Anxiety and Depression Scale. Correlation and hierarchical regression analyses were applied to understand the clinical and behavioral correlates of DE/IR behavior and its association with anxiety and depression.

**Results:**

Among the 110 patients with type 1 and type 2 DM recruited, we found 17.6% restricting insulin use and 6.3% self-medicating for weight control (higher in type 2 DM than type 1 DM). Hierarchical regression analyses showed HbA1c standard deviation (odds ratio = 2.18, [95% confidence interval 1.07–4.42]), body image (1.83, [1.05–3.20]), and dieting (4.74, [1.70–13.23]) associated with DE/IR behavior. Moreover, DE/IR behavior was further associated with anxiety (1.17 [1.08–1.27]) and depression (1.12 [1.03–1.22]).

**Conclusion:**

DE/IR behavior is not uncommon among AYAs with DM, particularly those with type 2 DM, and may be associated with anxiety and depressive symptoms. In addition, HbA1c variability is correlated with DE/IR behavior, and the clinical implications need further exploration.

**Supplementary Information:**

The online version contains supplementary material available at 10.1186/s40337-023-00888-8.

## Abstract (highlights)

While young patients with diabetes mellitus (DM) are prone to eating disorders that may worsen metabolic control, early detection and appropriate intervention of comorbid emotional and behavioral symptoms are urged when providing diabetes care to this vulnerable age group. Having observed an increasing trend of eating disorders in the general population in Taiwan, we aimed to investigate clinical and behavioral correlates of disordered eating and insulin restriction behavior and its association with psychological health in a clinical sample of youths with DM. We found that 17.6% of the young patients with DM restricted insulin use, and 6.3% self-medicated for weight control. Fluctuating glucose levels and body image issues were found to be correlated with disordered eating and insulin restriction behaviors, which were also significantly linked to risks for anxiety and depression. These findings may warrant scrutiny in assessing eating behavior and insulin use among young patients with DM in East Asian settings.

**Supplementary Information:**

The online version contains supplementary material available at 10.1186/s40337-023-00888-8.

## Introduction

Type 1 diabetes mellitus (T1DM) accounts for 10–15% of all diagnosed cases of diabetes each year, and its incidence is increasing with time [1]. Managing T1DM is a complex process that requires strict adherence to a structured plan, including appropriate nutritional management, prescribed pharmacotherapy, regular blood sugar monitoring, and regular physical exercise [2]. Patients with T1DM are at risk for disordered eating and eating disorders due to specific patterns and featured management, such as insulin-related weight gain and diet for hypoglycemic prevention [3]. On the other hand, type 2 DM (T2DM) manifests as hyperglycemia that usually ensues as a consequence of excessive body fat and insulin resistance [4]. Disordered eating, particularly binge eating behavior, is not uncommon in patients with T2DM [5]. Worth clinical attention, their presenting symptoms may differ from those commonly seen in non-diabetic eating disorders [5, 6].

Restricting insulin against dosing instructions provided by doctors is a readily available method for weight control [7]. However, this method warrants clinical attention in patients with T1DM because it may increase the risk of eating disorders and metabolic problems leading to an increased risk of diabetic complications, such as retinopathy, neuropathy, and hospitalization for diabetic ketoacidosis [6]. Deliberate insulin reduction or omission has been associated with recurrent hypoglycemia and hyperglycemia with diabetic ketoacidosis events that are further associated with elevated mortality risks [8]. Moreover, diabetes treatment associated with insulin use and adolescent weight changes may also increase the incidence of persistent eating problems [9]. Additionally, adolescence is considered a sensitive period that is a risk factor for disordered eating behavior, which is seen at a higher rate in patients with T1DM and T2DM than in their peers without diabetes [4, 10]. While inappropriately restricting insulin has been extensively investigated for its association with poorer health in individuals with T1DM, less has been researched on the insulin-treated counterparts of T2DM, where they need insulin injections as their primary treatment because they fail to reach their glycemic goal with other hypoglycemic medications [11–13].

There is substantial evidence supporting a correlation between mental health issues and poor glycemic control in adolescents and young adults (AYAs) with DM [14–16]. Early detection and appropriate intervention of comorbid emotional and behavioral symptoms are therefore urged when providing diabetes care to this vulnerable age group. However, empirical data on psychobehavioral issues, such as disordered eating and insulin restriction (DE/IR) behaviors, among AYAs with DM is relatively scarce in East Asian social settings. One prior study a decade ago found that Taiwanese adolescents with T1DM exhibited more disturbed eating behaviors than their adolescent counterparts without diabetes, but it did not investigate IR practice [17]. Having observed an increasing trend of eating disorders in general Taiwanese AYAs in recent years [18], we aimed to investigate the clinical and behavioral correlates of DE/IR behavior and its association with psychological health in a clinical sample of AYAs with DM.

## Methods

### Study subjects

**Fig. 1:**
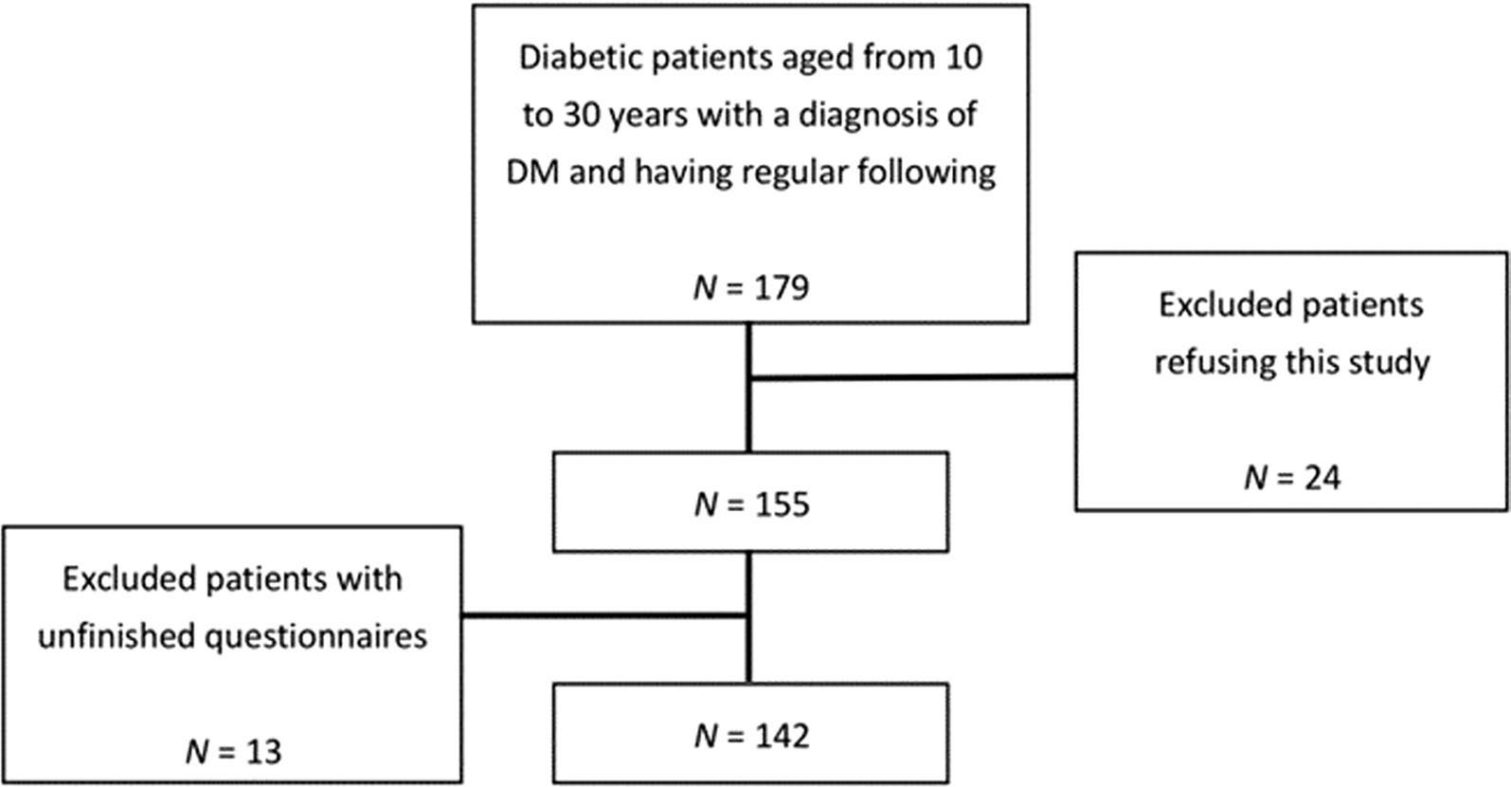
The flowchart for the selection of patients; DM: diabetes mellitus

We recruited patients with T1DM and T2DM aged 10–30 years, who received the diagnosis before age 18 years and were regularly tracked in the pediatric outpatient clinic at a single medical center that received referrals from a catchment area of nearly 3 million residents in southern Taiwan [19]. A total of 179 cases receiving insulin as their primary treatment were initially accessed, and 24 chose not to participate, leaving 142 patients for analysis (Fig. 1). In routine clinical practices in Taiwan, we tested for islet autoantibodies and performed the glucagon stimulation test to distinguish between T1DM and T2DM in cases who needed basal-and-bolus insulin as their main therapy, because patients with T1DM received a catastrophic illness status eligible for partial medical fee waiver. Those with negative islet autoantibodies (i.e., glutamic acid decarboxylase 65 antibodies) and an appropriate C-peptide level (i.e., 0.7 nmol/l for fasting and 1.1 nmol/l for post-glucagon C-peptide levels) in response to glucagon stimulation were deemed T2DM [20]. This study was approved by the Institutional Review Board of the Cheng Kung University Hospital (A-BR-107-033).

### Collection of clinical data

We reviewed medical charts to obtain relevant clinical data, including gender, type of DM, disease duration, body mass index (BMI) at onset and present, and insulin dosing. BMI was calculated by dividing a patient’s weight in kilograms by their height in square meters. We determined the *z*-score of BMI according to gender and age-specific BMI charts using the Taiwan children and adolescent growth chart published in 2010 [21]. Multiple visit-to-visit hemoglobin A1c (HbA1c) levels were recorded over one year prior to the enrollment date. Aligned with previous research, we calculated the mean, standard deviation (SD), coefficient of variation (CV), and visit-to-visit variability score (VS) of the HbA1C levels to reflect patient glycemic control and fluctuation [22, 23]. In addition, we calculated the insulin dose-adjusted A1c (IDAA1c) according to the following formula: HbA1c (%) + 4 × insulin dose (units/kg/day). Using both total insulin dose and HbA1C in the same formula can reduce the influence of the treatment regimen when considering glycemic control [24].

### Eating behavior

We used two specific questionnaires to obtain the eating behavior among AYAs with DM: the 21-item revised Three-Factor Eating Questionnaire (TFEQ-R21) and the modified SCOFF (mSCOFF) questionnaire. The TFEQ-R21 has been validated for evaluating eating behavior among adolescents in three aspects, including emotional eating (EE), uncontrolled eating (UE), and cognitive restraint (CR) [25]. The original SCOFF questionnaire is a reliable and valid screening instrument encompassing five dichotomous items (i.e., intentional vomiting, loss of control over food, unhealthy weight loss, body image disturbance, and intrusive food thoughts), and its Mandarin version has been validated in a Taiwanese setting [26]. Like the original SCOFF score reflecting the number of disordered eating behaviors, with a score of 2 or greater considered a risk for the occurrence of eating disorders [27], the mSCOFF questionnaire replacing the final item (intrusive food thoughts) with insulin restriction with the question “Do you ever deliberately take less insulin than you should?” has been proposed for the young population with diabetes because adolescent patients may deliberately adopt insulin restriction as an alternative behavior for weight control [28, 29]. In this context, patients might restrict insulin doses inappropriate to carbohydrate intake against their doctors’ dosing instructions. The cutoff of 2/5 on the mSCOFF questionnaire may suggest DE/IR behavior with a decent index of sensitivity (80%) and specificity (90%) as compared to the Eating Disorder Inventory-3, and a score above the threshold therefore requires a thorough psychological interview in patients with diabetes [29]. For the above reasons, we used the mSCOFF questionnaire to evaluate DE/IR behavior.

### Body image and weight-control behavior

A single item was used to ask how the participants viewed their body size. The answers were rated on a 5-point Likert-like scale from very thin (score = 1) to very heavy (score = 5) [30]. Moreover, we screened five different types of weight control behavior (i.e., restricting insulin use, dieting, self-medicating weight-loss or laxative pills, induced vomiting, and over-exercising) in the past three months using dichotomous questions.

### Psychological wellbeing

We used the Hospital Anxiety and Depression Scale (HADS) to measure participants' psychological wellbeing in two domains (i.e., anxiety and depression), and each domain had seven items rated using a four-point Likert-type scale [31]. The psychometric properties of the HADS Mandarin version were supported in Taiwanese youth [30]. After reverse coding the negatively worded items and adding up all the item scores, higher scores on the HADS represents higher levels of anxiety and depression. In the present analysis, a domain score of 11 or greater indicates risks for anxiety or depression [31, 32].

### Statistical analysis

We summarized the clinical, behavioral, and psychological variables using descriptive statistics and compared these variables between patients with T1DM and T2DM using Student's t and Chi-square tests as appropriate. A Pearson correlation analysis was applied to examine the bivariate correlation between psychological and behavioral variables of interest. Firstly, we used univariate and multivariate logistic regression analyses to identify potential clinical and behavioral correlates of DE/IR behavior, defined by an mSCOFF score of 2 or greater. Further, we used hierarchical regression analyses, recursively controlling for clinical and behavioral confounders, to evaluate the effects of DE/IR behavior on psychological wellbeing. Specifically, Model 1 tested the univariate association between mSCOFF scores and anxiety and depression. Model 2 controlled for behavioral parameters. Model 3 controlled for clinical and behavioral parameters. Covariates, including age, gender, and types of DM, were included in all models. A stepwise predictor selection was used with a significance level of 0.05 for entry and 0.1 for stay in the multivariate analyses. Odds ratios (ORs) with a 95% confidence interval (CI) were reported for the predictors remaining in the final model.

## Results

Table 1 describes the demographic and clinical parameters of the patients with DM (N = 142) at the time of data collection. Among them, 110 (77.5%) had T1DM, and the rest had T2DM and used basal-and-bolus insulin as their primary treatment because of unsatisfied diabetic control with other hypoglycemic medications. There was no difference regarding age at enrollment or gender between T1DM and T2DM. However, the disease duration was longer among patients with T1DM (9.41 ± 6.23 years) than those with T2DM (2.94 ± 3.66 years). The mean BMI *z*-score at enrollment was lower among T1DM (0.52 ± 1.55) than T2DM (2.99 ± 2.71) patients. There was a higher mean HbA1c and IDAA1c in patients with T1DM than those with T2DM. Conversely, HbA1C-SD and HbA1C-CV were lower in patients with T1DM than those with T2DM. Moreover, patients with T2DM had a greater concern for body image and were more likely to use weight-control medications or restrict insulin use. Stratifying the patients by weight status, we observed a trend that patients who had overweight/obesity tended to have more DE/IR behaviors (Additional file 1: Table S1).

**Table 1:** The demographic and clinical parameters of the patients stratified by type 1 and type 2 diabetes mellitus

	T1DM (n = 110)	T2DM (n = 32)
Age, mean (SD)	17.70 (5.05)	16.19 (4.14)
*Gender* Male	50 (45.5%)	16 (50%)
Female	60 (54.5%)	16 (50%)
Duration (years), mean (SD)	9.41 (6.23)	2.94 (3.66)*
BMI Z score now, mean (SD)	0.52 (1.55)	2.99 (2.71)*
BMI Z score onset, mean (SD)	0.08 (1.36)	3.00 (2.79)*
HbA1c, median (25th–75th percentile)	8.05 (7.48–9.23)	7.63 (6.28–8.64)*
HbA1c-SD, mean (SD)	0.64 (0.57)	1.05 (0.94)*
HbA1c-CV, mean (SD)	0.07 (0.06)	0.13 (0.10)*
HbA1c-HVS, mean (SD)	47.58 (28.05)	55.73 (34.22)
IDAA1c, mean (SD)	13.47 (2.83)	10.71 (2.08)*
TFEQ-R21, mean (SD)	2.05 (0.39)	2.08 (0.49)
EE	1.91 (0.73)	1.94 (0.86)
UE	2.07 (0.51)	1.97 (0.54)
CR	2.18 (0.51)	2.38 (0.54)
Body image, mean (SD)	3.63 (0.97)	4.13 (0.98)*
Restricting insulin (%)	11%	45.5%*
Dieting (%)	20.9%	25.8%
Self-medicating (%)	3.6%	15.6%*
Induced vomiting (%)	0%	0%
Over-exercising (%)	5.5%	15.6%
mSCOFF, mean (SD)	0.84 (0.93)	1.45 (1.37)
HADS anxiety, mean (SD)	0.98 (0.41)	0.88 (0.49)
HADS depression, mean (SD)	0.75 (0.45)	0.55)

*BMI* body mass index, *T1DM* type 1 diabetes mellitus, *T2DM* type 2 diabetes mellitus, *HbA1c* glycated hemoglobin, *HbA1c-SD* HbA1c-standard deviation, *HbA1c-CV* HbA1c-coefficient of variation, *HbA1c-HVS* HbA1c-variability score, *IDAA1c* insulin-dose adjusted A1c, *TFEQ-R21* three-factor eating questionnaire-R21, *EE* emotional eating, *UE* uncontrolled eating, *CR* cognitive restraint, *mSCOFF* modified SCOFF eating disorder screening questionnaire, *HADS* hospital anxiety and depression scale, *SD* standard deviation

*p < 0.05

The Cronbach's alpha values for the questionnaires employed in our study were acceptable (ranges: 0.62–0.93), except the one for the mSCOFF, which was only 0.41 (Table 2). In bivariate correlation analysis, scores on the mSCOFF were correlated with those on the CR subscale of the TFEQ-R21, both the depression and anxiety subscales of the HADS and body image. Moreover, scores on the EE and UE subscales of the TFEQ-R21 were correlated with those on the anxiety subscale of the HADS. Scores on body image were also correlated with those on the depression and anxiety subscales of the HADS. In univariate regression analysis, we found that several factors, including TFEQ-R21 CR (OR = 2.37, [95%CI 1.04–5.40]), body image (OR = 2.07, [95%CI 1.25–3.44]), dieting (OR = 6.48, [95%CI 2.50–16.77]), and over-exercising (OR = 8.42, [95%CI 1.51–46.85]), were associated with an mSCOFF score of 2 or greater (Table 3). However, in the full multivariate regression analysis, only HbA1c-SD (OR = 2.18, [95%CI 1.07–4.42]), body image (OR = 1.83, [95%CI 1.05–3.20]), and dieting (OR = 4.74, [95%CI 1.70–13.23]) were associated with an mSCOFF score of 2 or greater.

**Table 2:** Validation and correlation analysis on the employed questionnaires

	mSCOFF	TFEQ-R21 EE	TFEQ-R21 UE	TFEQ-R21 CR	HADS-anxiety	HADS-depression	Body image
mSCOFF	1						
TFEQ-R21 EE	0.14	1					
TFEQ-R21 UE	0.1	0.56**	1				
TFEQ-R21 CR	0.26**	0.14	− 0.11	1			
HADS-anxiety	0.36**	0.27**	0.26**	0.15	1		
HADS-depression	0.24**	0.07	0.16	− 0.1	0.27**	1	
Body image	0.31**	0.10	0.05	0.23**	0.21*	0.17*	1
Cronbach's alpha	0.41	0.93	0.82	0.73	0.64	0.62	–

*mSCOFF* modified SCOFF eating disorder screening questionnaire, *TFEQ-R21* three-factor eating questionnaire, *EE* emotional eating, *UE* uncontrolled eating, *CR* cognitive restraint, *HADS* hospital anxiety and depression scale

*p < 0.05; **p < 0.01

**Table 3:** Univariate and multivariate regression analyses of the physiological and behavioral correlates with mSCOFF scores

	Univariate	Multivariate
*Clinical parameters*		
Disease duration	1.01 (0.95–1.08)	
BMI Z-scores	1.23 (0.99–1.52)	
HbA1c (Group)	1.19 (0.68–2.07)	
HbA1c-SD	1.84 (0.98–3.45)	2.18 (1.07–4.42)*
HbA1c-CV (%)	1.06 (1.00–1.12)	
HbA1c-HVS	1.01 (1.00–1.02)	
IDAA1c	1.00 (0.86–1.15)	
*Behavioral parameters*		
TFEQ-R21 EE	1.24 (0.73–2.11)	
TFEQ-R21 UE	1.48 (0.66–3.31)	
TFEQ-R21 CR	2.37 (1.04–5.40)*	
Body image	2.07 (1.25–3.44)*	1.83 (1.05–3.20)*
Dieting	6.48 (2.50–16.77)*	4.74 (1.70–13.23)*
Self-medicating	2.01 (0.34–13.30)	
Over exercising	8.42 (1.51–46.85)*	

*BMI* body mass index, *HbA1c* glycated hemoglobin, *HbA1c-SD* HbA1c-Standard deviation, *HbA1c-CV* HbA1c-coefficient of variation, *HbA1c-HVS* HbA1c-variability score, *IDAA1c* insulin-dose adjusted A1c, *TFEQ-R21* three-factor eating questionnaire-R21, *EE* emotional eating, *UE* uncontrolled eating, *CR* cognitive restraint, *mSCOFF* modified SCOFF eating disorder screening questionnaire

*p < 0.05

Further, in an attempt to examine the association between DE/IR behavior and anxiety and depression based on the HADS questionnaire, we found that mSCOFF scores were consistently associated with depression and anxiety based on the HADS questionnaire, even after controlling clinical and behavioral parameters (Table 4). In the fully adjusted model, an mSCOFF score of 2 or greater was associated with a 17% increase in the OR for anxiety and a 12% increase in the OR for depression based on the HADS questionnaire.

**Table 4:** The hierarchal regression analyses of the association between mSCOFF scores, behavioral and clinical parameters, anxiety and depression

	Anxiety			Depression		
	Model 1	Model 2	Model 3	Model 1	Model 2	Model 3
mSCOFF	1.19 (1.09–1.28)*	1.17 (1.09–1.26)*	1.17 (1.08–1.27)*	1.12 (1.04–1.22)*	1.12 (1.03–1.21)*	1.12 (1.03–1.22)*
*Behavioral parameters*						
TFEQ-R21 UE		1.17 (1.03–1.35)*				
TFEQ-R21 CR					0.85 (0.73–0.99)*	0.83 (0.70–0.97)*
Over-exercising					1.39 (1.05–1.86)*	1.49 (1.06–2.12)*
*Clinical parameter*						
HbA1c-HVS			1.01 (1.00–1.01)*			

*HbA1c-HVS* HbA1c-variability score, *TFEQ-R21* three-factor eating questionnaire-R21, *UE* uncontrolled eating, *CR* cognitive restraint, *mSCOFF* modified SCOFF eating disorder screening questionnaire

Only significant associated parameters were listed

*p < 0.05

## Discussion

To the best of our knowledge, this paper is the first to investigate psychosocial and metabolic correlates of insulin restriction among AYAs with DM in Taiwan. We observed that patients with T2DM were more likely to have body image concerns and adopt medications and inappropriate insulin restriction against their doctors’ dosing instructions as weight-control measures. Moreover, DE/IR behavior was associated with psychological distress, such as anxiety and depression based on the HADS questionnaire, which requires clinical attention when consulting these patients in practice.

In this study, we found that 11% of the patients with T1DM had at one point restricted their insulin use. The prevalence was slightly lower than those reported in Western societies [3, 33]. This prevalence may correspond to a lower prevalence of eating disorders in an East Asian social setting [18]. However, nearly half of the patients with T2DM who need basal-and-bolus insulin as their treatment strikingly reported inappropriately restricting insulin use against their doctors’ instructions (i.e., deliberately taking less insulin than required), and a greater proportion of body image concern and disordered eating was seen in the patients with T2DM than those with T1DM. Marked weight gain may ensue if insulin doses are too high or incorrectly distributed when using insulin therapy in T2DM [34]. In our questionnaire, the item explicitly indicated that young patients adjusted insulin doses because of fear of weight gain rather than changes in carbohydrate intake. In adults with T2DM, the reasons for refusing insulin can be attributed to psychological factors, such as fear and a negative perception of insulin, as well as cognitive factors, such as questioning the efficacy of insulin and seeking insulin-free therapy (e.g., GLP-1 receptor agonists) [35]. Further, refusing insulin may be attributable to lifestyle changes caused by psychosocial, peer pressure, and family-related factors that affect the quality of care in youths with T2DM [36]. As early-onset T2DM is more likely to be associated with greater risks of cardiovascular diseases and diabetic complications than adult-onset T2DM and T1DM [22, 36], how the insulin restriction behavior is related to the cardiometabolic outcomes should be carefully investigated among youths with T2DM who usually receive less attention than their peers with T1DM.

The mSCOFF has a similar level of internal consistency to the original SCOFF, which has been widely used in clinical practice and has acceptable psychometric properties for Chinese adolescents [37, 38]. Moreover, we found that mSCOFF scores were correlated with those of the CR subscale of the TFEQ-R21, both subscales of the HADS, and body image, indicating its external validity as a crucial psychosocial assessment to screen eating psychopathology. Using a cutoff mSCOFF score of 2 or greater to define DE/IR behavior, we did not find any significant association between DE/IR behavior and HbA1c levels or BMI. Our results are somehow inconsistent with those in Hsu et al.’s study [17], where BMI and HbA1C were found to be associated with the severity, but not symptoms, of bulimia on the self-report Bulimic Investigatory Test, Edinburgh (BITE), and only BMI significantly predicted oral control and dieting subscales on the Eating Attitude Test-26 (EAT-26). However, BITE and EAT-26 did not capture insulin restriction behavior, and this discrepancy in survey modalities may explain the inconsistency in findings. Despite so, we found a significant association between DE/IR behavior and HbA1c-SD, extending some more evidence that DE/IR behavior may have clinical relevance to stability in glycemic excursion in these patients. Further, in the hierarchal regression analyses, DE/IR behavior was consistently associated with depression and anxiety based on the HADS questionnaire, while BMI and metabolic parameters were not. These findings aligned with those found in a recently published paper on a Chinese cohort of T1DM youths and adults [39] and may suggest DE/IR behavior plays a central part in the interrelationships among body image concerns, externalizing and internalizing behaviors, and diabetic control. In another qualitative research paper, the authors identified low compliance to insulin intake among other psychological factors (e.g., fear of gaining weight) related to eating problems in Malaysian adolescents with T1DM via screening questionnaires and in-depth interviews [40]. Taken together, DE/IR behavior appears to be an essential indicator of multifaceted psychosocial challenges that AYAs with DM may encounter along with their diseases [41]. Healthcare providers for AYAs with DM should be aware of these counseling needs and integrate them into routine assessments, such as the mSCOFF, in addition to physical and biochemical check-ups [3].

Some limitations warrant attention when interpreting the results of this study. First, the analysis was cross-sectional, and thus the direction of causality may not be determined. Despite so, our findings on the association between DE/IR behavior and depression and anxiety based on the HADS questionnaire suggest the worth of vigilant monitoring of psychological and behavioral wellbeing among AYAs with DM. Meanwhile, a longitudinal and prospective follow-up is needed to address this issue. Second, the lack of reporting of oral medications in patients with T2DM may affect the estimates for clinical parameters of glycemic control. Third, the cohort was mainly derived from a single tertiary referral center, thus limiting the generalizability of the results. Also, due to a limited number of patients mixed with T1DM and T2DM and a wide range of ages from children aged 10 to adults aged 30 years, we were therefore unable to stratify our analysis by DM types. A multicenter registry of young patients with DM is needed to thoroughly investigate the occurrence and correlates of DE/IR behavior in the local population with specific regards to DM types and age groups.

## Conclusion

In a social context with a relatively lower prevalence of eating disorders, DE/IR behavior is not uncommon among AYAs with DM using insulin as their primary treatment. Given its relevance to psychological and glycemic outcomes, DE/IR behavior should be meticulously screened in health care provided to AYAs with DM. Appropriate psychological support and nutritional and dietary guidance are needed to ensure their healthy trajectory.

### Supplementary Information

**Additional file 1. **Table S1.

## Data Availability

The study does not have ethical approval to share data.
