# Supplementary material for: Correlates of disordered eating and insulin restriction behavior and its association with psychological health in Taiwanese youths with diabetes mellitus
Source: J Eat Disord. 2023 Sep 14;11:158. doi: 10.1186/s40337-023-00888-8 (PMC10503123; doi:10.1186/s40337-023-00888-8)
Supplement: Supplementary file 1 — Additional file 1. Table S1. [file 40337_2023_888_MOESM1_ESM.docx]

| Supplementary Table 1. The mSCOFF scores stratified by weight status | | |
| --- | --- | --- |
|  | mSCOFF ≧2 | % |
| Weight status group |  |  |
| Underweight (n=3) | 0 | 0 |
| Normal weight (n=74) | 14 | 18.9 |
| Overweight/obesity (n=65) | 23 | 35.4 |

Note: Chi-squared test *p* value = 0.02 (95%CI: 1.18-5.26)
